# On Seasons Attending Epidemics

**Published:** 1808-04

**Authors:** J. Wood

**Affiliations:** Newcastle


### *To the Editors of the Medical and Physical Journal.*

GENTLEMEN,

I Lately sent you *one part* of an intended communication, and I now beg leave to offer you the other part of it.

As nothing can be more interesting to the medical enquirer and observer, that reports of the prevailing diseases in the different parts of the kingdom, so also I think nothing can more contribute to the improvement of medical science, than such reports, when accompanied with useful practical observations and remarks. Few years have passed in which I have not had opportunities of making observations of singular epidemic affections, but the last year has also been productive of the opportunity of observing several diseases that are spread by contagion. In attempting a report, I will, for the sake of a clear arrangement, first, notice the diseases of the last mentioned description; then the particular epidemics that prevailed; and, lastly, I will add an account of some of the most singular chronic diseases, that have occurred in my practice. Allow me first to premise, that the idea of attempting such a report, first arose from the satisfaction I have derived from similar reports* from different parts of the kingdom, by observing in them the same exemption, or the same prevalence;

* Particularly by Dr. Fothergill in your Journal, and lately by Mr. Roberton of Edinburgh.

lence, of those diseases which commonly derive their origin, in the first instance, from a particular state of the weather, (this has been uniformly the case for many years with respect to typhus fever, and has consequently led in my mind to almost certain conclusions on the subject;) and that with respect to those diseases, which are communicated by contagion, the reverse of the above remark generally takes place, as will appear from a comparison of the prevalence of such diseases in London, Edinburgh, and Newcastle, at the same time. Scarlet fever was introduced into this town (from the neighbourhood where it had raged for some months) in the autumn of 1806, but fortunately it was then prevented from spreading by the early detection of the disease in the persons of such as could be removed to the house instituted for the reception of contagious fever. The disease did not appear again until the end of May, when it was brought by several children from the neighbourhood where it still existed, and soon spread with a great rapidity; it has raged here from that time in the most general way, not only going through families of children, but also attacking the parents of such who had not previously undergone the complaint; and even, in several instances, adults, who had previously had the disease, were affected with a sore throat, that appeared to arise from the infection of scarlatina, to which they were much exposed.

As far as my observation enables me to speak, I would say, that adults who have formerly gone through the disease, are not a second time liable to the fever and eruption, but that they are sometimes affected with sore throat in consequence of being much among the infected. I have only once seen the disease in its full form *a second time* in the same person, and this occurred to a young lady, whom I attended some years ago, who had gone through the disease rather severely; and after an interval of three weeks, being then in the state of convalescence, was again attacked with it in nearly as severe a manner as before. The symptoms were all present, only less in degree, and went through the same course. This disease has, during the last two months, very much subsided; but only I think from its having no more objects to attack, to which it can get access; it has spread like fire, attacking every combustible substance to which its force could reach.

Chin-cough appeared here nearly about the same time, or rather a few weeks before scarlatina, and has also now almost disappeared; it was also very general, and widely spread.

spread. Measles made their appearance at a late period; at least, were not very general until the autumn months, when, indeed, they prevailed to an equal extent with either chin-cough or scarlet fever. Towards the end of the year, when the very cold weather took place, several cases of croup occurred. We have not been visited by small-pox; indeed, the inoculation for cow-pox is now so general here, that there would not be much to fear from the appearance of small-pox, I trust. If cow-pox has yet any prejudices to overcome in this neighbourhood, they are chiefly to be found in that rank of life where we should least expect to find them, I mean in the upper ranks; as to these, education has not only prepared the way for liberal inquiry, but information also finds more easy access; and although the lower ranks have not such advantages, yet the testimony of their neighbours, and that of their own senses, well supplies the place of general information; and prejudice, if ever it arose, was soon borne down by the popularity of the cow-pox, which popularity of course sprung, not from abstract reasoning, but from downright conviction. The inoculation of the cow-pox, at the Dispensary here, has been equally progressive, as when I last reported to you of it, and equally successful. We have not one unfortunate; not one suspicious case to record; not one instance of its failure. It has sometimes here excited surprise and remark, that the supposed instances of the failure of cow-pox to prevent the small-pox have almost all occurred in the south, and either in the metropolis, or near it.

When we look then to the reports for the last year, of the diseases spread by contagion, we will find that small-pox has committed great ravages in London, and in Edinburgh, but that the disease has not reached this place; that scarlet fever has prevailed here to the greatest extent almost, only a little in London, and only rumours of it in Edinburgh. Chin-cough and measles have appeared in London, Edinburgh, and this neighbourhood; the former rather earlier here, and both to a much greater extent, than either in Edinburgh or London, especially measles. Croup, after the cold weather, appeared in Edinburgh, and in this place. From the chronic nature of chin-cough, several instances occurred of children labouring under it, being attacked with the symptoms of measles: under such a circumstance I observed, that the chin-cough resisted the measles for a short time, and then the fever and eruption very suddenly took place; during the appearance of the the the measles, the chin-cough was suspended; and I observed (with Dr. Fothergill I think as mentioned in his Report, or with Dr. Bateman in his Report in the Edinb. Journ.) that as soon as the measles were over, the chin-cough returned. There was much reason to dread the existence at the same time of two diseases, both of which affected the lungs, and both attended with a distressing cough, yet I saw no instance, nor have I heard of any bad effects from such coincidence. Fatal effects of such are mentioned in the reports from London and Edinburgh; indeed, the two diseases are said to have been very fatal *separately* in those places; whereas, though they seem both to have been more prevalent here, yet they also appear to have been in general much milder, especially the chin-cough, for several instances of measles, in a severe form, did occur.

The practical remarks I have to make on the treatment of these diseases will be as few as they are simple, but not the less important I hope for being so: in saying this, I particularly allude to the treatment of scarlatina. *Cold water* externally, and the *muriatic acid* internally, when the state of the throat required it, was all that was necessary. Magnesia, or some gentle aperient, filled up the practice. The disease appeared under all its various forms, even in the same family, one child of which would have the sore throat without much fever, and no eruption; and the rest of the family would all have the eruption with considerable fever; in such there was a slight affection of the throat at the conclusion of the eruption. But in whatever form it appeared, if the fever was considerable, *cold water* was the most effectual remedy; it was applied with a sponge to the whole surface of the body three or four times a day, and as often in the night, if the skin became hot. The relief afforded by it by removing intense heat, delirium, restlessness, and procuring sleep, was greater than I can describe; in a few words, I can say, that by means of cold water, we have the disease under the most *complete command;* some persons simply washed their children with cold water by means of a linen cloth whenever they became hot, and so clearly were the good effects of this perceived by the parents, and felt by the children, that I had not on any occasion ever to *urge* the repetition of it, as it was always voluntarily done, after the first direction for its use. For this we are indebted to the late Dr. Currie, strongly sanctioned by Dr. Gregory of Edinburgh. I confess I felt a little timid in one or two of the first cases in which I applied it in the autumn of 1806; but this this last year, after the former experience, I as fearlessly as successfully directed it. It is also remarkable, that in every instance within my knowledge of scarlatina being thus cured by cold water, none of the *sequelæ* of the disease took place; and, vice versa, every case of dropsy that I saw subsequent to scarlatina, occurred in those instances only, where washing with cold water had not been used; and farther I observed, that desquamation readily took place after the application of cold water, and that desquamation had not taken place in those cases in which the dropsical symptoms did. Any one who has directed cold water to the surface of the body in typhus fever, and witnessed its salutary effects, must have felt how natural it was, by an easy inference, to extend the remedy to scarlet fever; I am more surprised, therefore, that it has not been as generally adopted in the latter disease. If the names of Dr. Currie and Dr. Gregory are not sufficient to recommend and establish this practice in scarlet fever, I fear *my efforts* to do so will not have the desired effect; yet I cannot but feel, that by thus bringing *them* forward in a widely circulated Journal, I may be the means of making more generally known *a source of decided relief in the highest state of fever,* and thus extending, as it were, the power of affording such relief to many fellow-creatures suffering from fever in different parts of the globe.

With respect to the two other most prevalent diseases of the last year, Chin-cough and Measles, I have not much to offer; except a few instances of the latter, both diseases appeared in a very mild form; and whenever the breathing became short, accompanied with pyrexia in either, I found the strict antiphlogistic regimen, with emetics of ipecac. relieve the symptoms. The measles seem to me to require *more management* than *medicine*; and I never saw any appearance of the eruption, nor any alarming determination to the lungs, under the attentive precaution of not exposing the skin to cold air before the disease had completely terminated externally.

As to the remaining disease I mentioned, Croup, I can only speak in the most desponding manner; I am of opinion, that when the attack is *very severe,* all the usual means of relief are unavailing, although immediately applied; when the symptoms are less violent, and have existed twenty-four hours, our efforts to relieve the complaint are as hopeless; and that whenever the membrane is formed, which is often the case in a few hours, the expectoration of it by the most fortunate exhibition of remedies is very problematical*. In addition to the cases of acute croup that have come under my notice, I have seen one case of chronic croup, and one of chronic spasmodic croup, resembling that described by Dr. Miller; the case of simple chronic croup was the only one that terminated successfully. Emetics of ipecac. with a constant *warm* plaster to the trachea, were the remedies that proved of use.

On entering on the second division of the diseases proposed for this Report, viz. of those which owe their origin in the first instance to a particular state of the weather, I may again repeat that, from the Reports I have had access to from different parts of the kingdom, such diseases have commonly prevailed at the same time, and in the same degree in different places, varying a little perhaps according to local situation. For many years I have observed this with respect to Typhus fever; a comparative exemption from it having taken place for a length of time in every part of the kingdom. Accidental cases only of this fever had appeared in this town from the autumn of 1803 to that of 1806, when it prevailed in a more general way, and continued until the beginning of last year. During this time I admitted about twenty cases into the Fever Hospital of this town, being then the attending physician to that institution. With respect to the treatment of this fever, I have to observe, that I have neither given bark nor wine to any patient in it, since the year 1792; and that I have had no reason to regret any want of success from the omission of either.

In a publication at that time, and which I have alluded to in a former communication to you, I used the following words, "Having in repeated instances exhibited the bark in Typhus unsuccessfully, and it having been taken in many of those instances in the most advantageous manner, I naturally began to doubt of the efficacy of this medicine in Typhus, and to feel the moral impossibility of my ever again relying on its powers for the cure of that fever." This was introductory to a report of the success of a very different

* A case of croup occurred some time ago in my own family, in a boy of five years of age. Blood-letting, emetics, and blistering were all used within a few hours after the attack with evident relief; yet, in that short time the membrane had formed, and it was only by persevering in the use of emetics for three days that it was expectorated, and then with the greatest difficulty.

different mode of treatment; viz. by antiphlogistics internally and externally, particularly by a solution of nitrate of potash, and sometimes by the nitric acid, and by washing with cold water. And at the conclusion of the same publication, I used words to the following effect, that "the (then) present common and general method of cure in Typhus (or by bark and wine, &c.) was very precarious, and not founded on any just or rational ideas of the nature of the disease." I had reason at this time to consider myself the first seceder from the established practice; and I will not even enquire whether I was or not, I will rest satisfied with soon finding myself in company with the late Dr. Currie, with Dr. Jackson, and lately with Dr. Hamilton, of Edinburgh, with Dr. Clutterbuck, and Dr. Beddoes; for, although the *means* used by each are *different*, yet it appears to me that the *same ultimate effect* is produced. I long ago shewed how the medical world had, in the treatment of Typhus fever, gone all at once from blood-letting to bark and wine, from one extreme to another; and I cannot but state my conviction, that we ought not to go back to the former extreme, as the desired effect, even according to Dr. Jackson's theory, may be obtained by other and safer means, such as the strict antiphlogistic regimen, ablution with cold water, and the nitric acid, or the nitric acid neutralised with potash internally.

A Catarrh, sudden in its attack, and violent in its form, was the next disease that prevailed here in the beginning of last year, as an epidemic, the effect of the change of the weather; it was so general, and the attending fever so sudden and severe, that it was called an Influenza; there was no suspicion of its being contagious, the usual means were sufficient for the cure of this Catarrh, though it exceeded common Catarrhs so much in degree.

About the last week in April, and the first week in May, a Catarrh of a different nature prevailed, confined entirely to the lungs, and suddenly producing symptoms of Phthisis. Out of 17 patients presented to me at the Infirmary here for admission on the same day, 15 were affected in this manner, and all dated their attack within a few days of each other. For some years past I have observed, that the foundation of Phthisis Pulmonalis had been laid about the first week of May, when in this neighbourhood very cold easterly winds prevail, with often at the same moment a hot sunshine.

Any view of the treatment of this disease would lead me far beyond the intended limits of this Report, the symptoms indicating the different species of it, and the different stages

 of of the same species, naturally leading to great variation in the means used for the cure, but something of this will come under another head. Before I quit the subject of Catarrh, I may mention, that it occurred very generally again in a less severe form about the first week in July; I was then in Berwickshire, where I first observed it, and on my return home I found many affected with it, and that it had affected almost every patient in our Infirmary at the same time. This leads me to remark, how common it is for every one to try to find out *how they get a cold*, not considering that it is often a general effect of the weather. At the same time, I do not mean to say, that such an affection may not easily be produced, independent of any general effect of the weather; and, vice versa, that by proper management it may often be prevented.

I come now to the most interesting epidemic which I observed last year; it was noticed by Dr. Fothergill, in your Journal for August, in a very particular manner, under the name of *Asthenia*. For the history of the symptoms, I cannot do better than refer to his Report; I will only briefly state a few of them here, to distinguish the peculiar affection, and add one or two that Dr. F. had not observed, with a notice of another disease that occurred in *affinity* with this extraordinary state of Asthenia.

This epidemic appeared about the second week in July, and the symptoms of it were not wholly relieved for three weeks. It began by a great sense of weariness, with that of fatigue on the least exertion, a desire to put off every, even necessary busines, weakness of the stomach with loss of appetite and difficult digestion, palpitation of the *aorta descendens*, with dyspnœa and intermitting pulse in consequence. It was rather a momentary *cessation* of the action of the aorta than a palpitation of it, but which frequently took place, especially in the afternoon; the dyspnœa that followed this cessation of the action of the aorta may easily be conceived, as much so as the intermitting pulse. It was remarkable, that this disagreeable affection in the circulation was not troublesome in the recumbent posture, the nights therefore were comfortable, and bed desirable. At this time a train of circumstances led me to think that the earth was very much deprived of its electricity, and that it was the deficiency of this powerful agent in the animal œconomy which produced the symptoms and state of Asthenia above described; and I believe this opinion, though not capable of demonstration, is nearly proved by a chain of collateral evidence. I do not recollect whether I was led led to this train of thinking by reading the remarks of Mr. Cornelius Varley, in your Journal for May last, (amongst the articles of Intelligence in that Number) as to the formation of clouds, &c. or whether those remarks strengthened my opinion; but this opinion is perfectly conformable not only to those remarks, but to every common observation, viz. that in fine, that is, in hot and dry weather, the earth must be giving electricity to the atmosphere by means of vapour, which is only *partially* returned by means of the same medium, and not completely restored but by means of rain or lightning. Such was the state of the earth at the time I have mentioned; and the epidemic affection I have described was somewhat relieved by a thunder storm, but not totally, until we had rain for many hours, on the 31st of the month. The symptoms being less felt in a morning, when the electrictity had been partially supplied during the night, and increasing towards evening, when the electricity was again exhausted by means of the heat of the day, as well as perhaps by that portion in our bodies being exhausted by exercise, &c. are circumstances strongly corroborating this theory; especially when we find that the epidemic was relieved by a thunder storm, and cured by the *first rain.* The symptoms being relieved by the recumbent posture*, is also in support of the theory, as *less vital power* is necessary to carry on the circulation in *that posture* than in the erect. During the state of the weather producing this epidemic, *spasmodic asthma* appeared in a few instances, and became worse in a patient who had lately recovered from a long attack of it. A medical friend of mine informed me, that he was seized with asthma in the evening when returning home from a journey in his carriage, and that he had not had an attack of it for many years; he was relieved by inhaling the vapour of concentrated vinegar. When therefore we consider what share oxygen has in producing the galvanic power, one modification of electricity, *and go round the circle,* we would be led to suppose that electricity itself is oxygen modified, and thus account for the relief my medical friend experienced from the *vapour of vinegar,* which too may be a likely method of supplying the system with a power simi-

* This observation has since led me to enquire into this circumstance in every palpitation, as it will assist in forming a diagnostic between palpitation from some particular state of the nerves, from palpitation arising from organic disease.

lar to electricity. It is remarkable, that hysteria was not prevalent during the epidemic I have described; I would therefore infer, that it proceeds from an opposite state of the nerves, the same as chorea arises from; chorea invariably taking place only before puberty, and hysteria after it in general, the change in the state of the uterus changing the form of the disease; and this seems probable farther from the very circumstance, that although hysteria may appear in young women before the *usual marks* of puberty, chorea on the other hand, never appears after *these* have taken place. To explain my *general theory* on this subject, as I promised in my last communication, when I mentioned the opposite methods of curing chorea by purgatives, according to Dr. Hamilton, and by the common mode of tonics, I suppose there exists in the nerves a *capacity* for the nervous power, and that this *capacity* may be diminished or increased; it may be diminished or *totally destroyed* by certain preparations of *lead*, according to the dose. This capacity may be diminished when *exceeding* the healthy state, by tonics, such as bark, cold bathing, &c. it may be increased when *below* the healthy state (as when it has been diminished by lead) by mercury; or when from other causes, by electricity, &c. When this capacity is totally destroyed, it often is never restored; for, in chorea, I suppose this *capacity* is increased, and the disease we see is in consequence of too much nervous power producing involuntary motion, not being under the command of the *will*. It is therefore evident you may either cure the disease by lessening this capacity, by means of the cold bath, tonics, &c.; or it may be purged off, perhaps, according to Dr. Hamilton's plan. This theory will also explain how chorea sometimes runs into the paralytic state, as the nervous power having accumulated from the increased *capacity* of the nerves, reacts on this capacity of the nerves and gradually destroys it, thus going *round the circle, from paralysis* proceeding from a sedative cause destroying the capacity, *to paralysis produced* by the *excessive action* of the nervous power itself *on the capacity of the nerve*.

Having extended this Report to so great a length, I dare say you will readily excuse my entering at present on the third proposed division of it.

I am, &c.

*Newcastle, February* 18, 1808. J. WOOD.

P. S. I have omitted to observe, that the patient I mentioned, who had a return of asthma during the period of a negative state of electricity, as I conjecture, was quite relieved that day on which it first rained for a few hours, and had not any return of the complaint; the debility inducing the disorder must be supposed to have been increased by the particular state of the air in this case. It is worthy of remark too, that a very moist air, after long hot and dry weather will restore electricity to the earth, in a sufficient length of time, without rain, and thus prevent the necessity of a thunder storm previous to rain; this is often effected by a moisture brought by an easterly wind, and immediately previous to the negative state of electricity taking place to so great a degree as I suppose it did, the earth was partially supplied, and a thunder storm, I conceive, prevented by such a moist state of the air for two days, on the 10th and 11th of July, which terminated in showers, after a few days of most extraordinary heat and dryness. This moist state of the air also enables the clouds, according to Mr. Cornelius Varley's remarks, to let go their electricity and precipitate in rain.

